# Integrating Software Engineering Processes in the Development of Efficient Intrusion Detection Systems in Wireless Sensor Networks

**DOI:** 10.3390/s20051375

**Published:** 2020-03-03

**Authors:** Iman Almomani, Afnan Alromi

**Affiliations:** 1 Computer Science Department, Prince Sultan University, Riyadh 11586, Saudi Arabia; 2 Computer Science Department, The University of Jordan, Amman 11942, Jordan; 3 HABOOB Cybersecurity Incorporation, Riyadh 13315, Saudi Arabia; a.alromi@haboob.sa

**Keywords:** software engineering, wireless sensor networks, WSN, requirement engineering, intrusion detection system, IDS, LEACH, packet dropping, network lifetime, energy, IoT

## Abstract

Applying Software Engineering (SE) processes is vital to critical and complex systems including security and networking systems. Nowadays, Wireless Sensor Networks (WSNs) and their applications are found in many military and civilian systems which make them attractive to security attackers. The increasing risks and system vulnerabilities of WSNs have encouraged researchers and developers to propose many security solutions including software-based Intrusion Detection Systems (IDSs). The main drawbacks of current IDSs are due to the lack of clear, structured software development processes. Unfortunately, a substantial gap has been observed between WSN and SE research communities. Integrating SE and WSNs is an emerging topic that will be expanded as technology evolves and spreads in all life aspects. Consequently, this paper highlighted the importance of Requirement Engineering, Software Design, and Testing when developing IDSs for WSNs. Three software IDS designs were proposed in this study: Scheduling, Broadcast, and Watchdog designs. The three designs were compared in terms of consumed energy and network lifetime. Although the same IDS approach was used, but, by highlighting the design phase and implementing different designs, the network lifetime was increased by 73.6% and the consumed energy was reduced by 20% in some of the designs. This is a clear indication of how following a proper SE process could affect the performance of the IDS in WSN. Moreover, conclusions were drawn in regard to applying software engineering processes to IDSs to deliver the required functionalities, with respect to operational constraints, with an improved performance, accuracy and reliability.

## 1. Introduction

Software engineering (SE) is an important discipline when developing software systems, especially in large-scale systems [1,2]. SE is concerned with all processes of software production. It is “a systematic approach to the analysis, design assessment, implementation, test, maintenance and reengineering of software” [1]. Thus, it is clear that the engineering of a software is an important problem-solving activity. SE ensures control over software functionalities, quality, and resources [1,2]. Hence, it ensures complete software development and requirement satisfaction. 

The urge of applying SE processes is of vital importance especially to large, complex systems including networking and security service systems [3,4,5,6]. Such systems are associated with risks that increase in unattended environments such as wireless networks. Therefore, embracing a holistic approach of any weakness along the engineering process of the system is required [1,2,3,4,5,6] to secure the networks from vulnerabilities that may lead to future security breaches. 

Security concerns have become particularly acute in systems deployed over wireless networks [7,8,9,10,11,12]. In recent years, advances in micro-electronic systems technology, digital electronics, and wireless communications that have enabled the development of Wireless Sensor Networks (WSNs) [6,13] have been witnessed. WSN is a self-organized network that consists of hundreds to thousands sensor nodes that are connected by wireless links [4]. These wireless sensors are small in size, low-cost, low-power, multi-functional, and communicate over short-ranges [4,5,6,14]. Sensor nodes have the capability of sensing, collecting, processing, and communicating in an autonomous manner [8,13].

WSNs are one of the most promising technologies and they have been embraced more than ever [6,8,15]. They have been used and deployed in different environments for different purposes, so their applications vary—for instance, military, habitat monitoring, disaster management, and environmental applications [8,11,16]. However, due to the distributed nature, cost, size, and power constraints of the sensor nodes, WSNs result in stringent limitations on node resources such as energy, computational speed, memory, and communication bandwidth. These limitations pose several challenges such as sensor battery lifetime, efficient distributed signal processing, data processing, and network security [13,16]. However, the two main and critical challenges are the sensors’ lifetime (i.e., period of operation) and the security of the network [11,17,18].

As for the lifetime of WSNs, sensor nodes use batteries as their power supply, but they are limited in their resources. For example, if sensor network is installed and deployed in a far geographical space to monitor physical phenomenon, they will be unapproachable [18]. Hence, recharging or replacing those sensor nodes will cost more because of the far distance.

As for the security of WSNs, owing to the network limitations, it is difficult to achieve security in applications deployed over WSNs [8]. These sorts of networks are usually deployed in remote and hostile environments to perform its tasks [4,12,19]. However, hostile environments are usually unattended. Due to that, WSNs lack physical protection (e.g., no switches or gateways to monitor the flow of information) resulting a potential of node compromising as well as low network security and protection [10,15,19]. Therefore, it is important to secure such networks from intrusions and attacks, especially in applications where security services are important. Efficient security mechanisms are in demand in order to be safeguarded and secured from attacks. 

As a first line of security defense, intrusion detection and prevention approaches can be used in order to reduce possible intrusions. Many types of attacks can be performed over a WSN, for example, Sinkhole Attack, Sybil Attacks, and Packet Dropping Attacks [20]. Among those attacks, packet-dropping attacks (i.e., packet loss) are one of the most disruptive and devastating threats in WSNs [21]. Packet-dropping attack drops the received data packets or control messages instead of forwarding them to other nodes, disrupting the normal operation of the network. 

Security attacks need to be conveniently addressed by detecting and preventing such malicious behaviors. WSN security has drawn the attention of many researchers [7,9,10,11]. To address and overcome the security issue which is considered one of the main challenges in WSNs, many security solutions were developed including software-based network Intrusion Detection Systems (IDSs) [3,6,9,12]. IDSs are classified according to different properties [9,16]. Therefore, these systems are deployed on different applications and environments. Accordingly, they have different SE requirements, designs, architectures, and implementation methods [1,2,3,4,22,23]. Consequently, developing a secure IDS while considering the energy resource is important. However, from [9,22], it can be seen that not all IDSs fulfill this aspect. Moreover, the development of such systems is unstructured and does not deploy the software engineering processes; therefore, this research was conducted. Many IDSs were developed, but not with the best designs that provides the best performances [9,21,24,25].

Building efficient IDSs for WSN is significant to ensure its secureness against different types of security attacks threatening its services. As part of the development of these IDSs is purely software-based, a clear software development life cycle (SDLC) should be identified and followed. This paper addressed the absence of following SDLC throughout the development of IDS for WSN. Moreover, this research studied the integration of software engineering process in the development of IDSs and how this affects the performance of WSN services. 

Therefore, this research developed an enhanced version of a hierarchical energy efficient IDS that was proposed by the authors in [21]. The development of the enhanced IDS has followed the SDLC phases. This study has focused mainly on the Requirement Engineering, Software Design, and Testing processes. One of the main thrusts of this research is to follow the SDLC phases in the development of the IDS and to show how this affects the overall performance of the IDS. In addition, three software designs were developed, tested, and compared in order to show the importance and the effect of different software designs. Such IDS development practice will contribute to the fields of SE as well as WSNs. 

Moreover, this study illustrated the most common mistakes that have been practiced when developing IDSs in relation to requirement engineering. As for the software testing, this paper discusses how the testing was carried out and based on what criteria and metrics the performance was measured. Additionally, the inconsistency in the adaptation of the performance measurements that have been used by various authors and researchers was also discussed, as there is no standardization or a common list of measures in the field of WSN. Moreover, after developing the IDS, it was tested and the test results of the enhanced IDS with the previous IDS were compared and then analyzed. The results revealed an increase in the network lifetime and reduction in the consumed energy when proper designs were implemented, although the IDS approach was not changed. This ensures the importance of following suitable SDLC while developing IDS for WSNs.

Apart from this introduction, the rest of the paper is structured into seven sections: Section 2 discusses the related work and highlights the innovativeness of this research, and Section 3 presents this research methodology and components. Section 4 discusses the proposed work including deep analysis of the current IDSs from SE perspectives and proposing an integration of SE process to the development of IDSs in WSN to enhance their performances. Section 5 goes through a case study and proposes three different designs. Section 6 illustrates the results and compares the three suggested IDS designs. Lastly, Section 7 consists of the conclusions, limitations, and future research directions.

## 2. Literature Review

WSN security has drawn the attention of many researchers [7,8,9]. Some research studies have focused on building trust and reputation in WSNs at different contexts and using different measures [26,27,28]. However, in the past few years, it has been witnessed [9,12,25,29,30] that IDSs have been developed and are very well investigated by researchers. However, those developed IDSs are not efficient enough to detect all malicious behaviors in a WSN. One of the main reasons is due to not deploying the SE processes.

**Figure 1:**
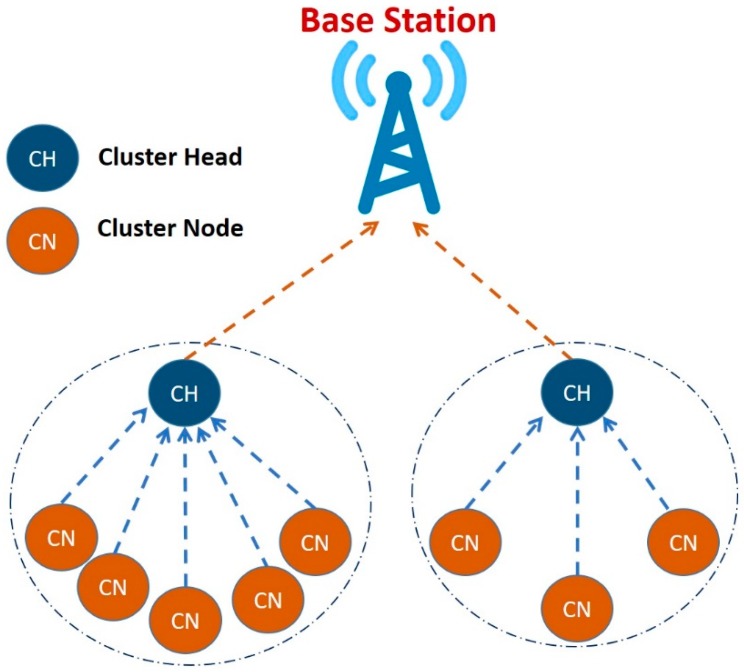
LEACH protocol topology.

Equally important, researchers and software engineers need to consider energy consumption when developing IDSs to defend attacks. One of the first energy efficient protocols for WSN is Low-Energy Adaptive Clustering Hierarchy (LEACH) protocol [23]. LEACH is defined as “a self-organizing, adaptive clustering protocol that uses randomization to distribute the energy load evenly among the sensors in the network” [24]. The LEACH’s routing process is as follows:

- The network is divided into a collection of clusters. Each cluster is managed by its cluster head (CH);
- Each CH has its own nodes, called Cluster Nodes (CN);
- The CH node sets up a time division multiple access (TDMA) schedule and transmits this schedule to all CNs;
- Then, the CNs transmit their data messages to their corresponding CHs;
- Afterwards, the CHs aggregate and compress the data and forward it to the base station (BS). This is because energy consumption of WSN can be reduced by allowing only CHs to communicate with the base station (BS). Figure 1 illustrates how the topology is built in LEACH protocol.

LEACH protocol was developed to increase the lifetime of WSNs; however, security was not considered [23]. Therefore, researchers have extended the LEACH protocol, with the attempt of securing its routing services [23,27]. Furthermore, not all extended LEACH protocols used in packet dropping detection systems are secured enough or energy efficient.

Some extended protocols have compromised the network energy because of their IDS mechanism—for example, the proposed IDS in [15] has introduced security to LEACH protocol through cryptographic algorithms. However, this approach has compromised the energy, since those types of algorithms require a lot of processing. 

To elaborate more on the IDS studies proposed by researchers, a sample of existing IDSs are explored and reviewed in this literature.

In [21], the authors have proposed a hierarchical energy efficient IDS for black hole attacks. The proposed detection schema has introduced a new layer to LEACH protocol called the second cluster head (SCH) layer. The selected SCH node keeps track of what has been received by the CH. The control packets contain the node identifier and the number of packets received by the CH. Then, the control packets are exchanged between SCH and BS, in order for the BS to compare the number of packets received from the CH and SCH.

Some IDSs do not only detect attacks, but they also remove them from the network to prevent the attack from happening again [31,32]. Furthermore, some researchers took different approaches when exploring and developing IDSs by considering the energy efficiency and simplicity. Energy efficiency, in IDSs, is as important as security because one of the main challenges of WSNs is the network’s lifetime [33] or, otherwise, the network would be useless. As mentioned earlier, LEACH protocol was developed to increase the lifetime of WSNs; however, security was not taken into account [34,35,36]. Therefore, the need for security in LEACH protocol has inspired many researchers to extend the LEACH protocol with the attempt of adding security features, to secure the routing process [34,35] and have it be resilient against insider and outsider attackers [36]. To elaborate more on the extended protocols used as IDSs, some studies are discussed below:

- In [15], S-LEACH was developed, and it was the first protocol that added security to LEACH. Two important security properties were added: data authentication and data freshness. Data authentication ensures the receiver that the data was really sent by the claimed sender. Data freshness ensures that the message was not a replay to an old message.
- In [37], SecLEACH was developed, and it was based on a random key distribution mechanism. The IDS enhanced the security of the S-LEACH through using a random key pre-distribution technique. In addition, it enhanced the Node-to-CH authentication but still it had some drawbacks. For example, data integrity of the schedule message was not delivered [35].
- In [18], MS-LEACH was developed and it was based on multi-hop/single-hop transmission. The IDS enhanced the security of S-LEACH IDS through providing node-to-CH authentication and data confidentiality using pairwise keys shared between CHs and their cluster members [18,35]. One of its drawbacks, it did not provide authentication for join request messages [35].

Moreover, from the performance evaluation provided in the literature and in [38,39,40], it can be seen how IDSs have compromised the network energy because of their IDS mechanisms. As some researchers have introduced security through cryptographic algorithms, where those types of algorithms require lot of processing causing the energy requirement to be compromised. So it can be concluded, that not all IDSs are secure enough nor energy efficient [22,25,39]. Furthermore, performance measurements and metrics were used in evaluating IDSs and routing protocols are reflecting the efficiency of the simulated network [41]. The used performance measurements and metrics differ from one study/research to another which include the network lifetime, number of rounds, consumed energy, delivered packets, delay and overhead measurements [40,42]. Certainly, what we are measuring and evaluating will make a difference in the performance selection criteria/metrics, either if it is for the purpose of measuring the energy efficiency, security, scalability or overhead. However, it has been witnessed from several studies in [21,43,44] that different performance measurements were used regardless of the purpose (and even for the same purpose). There is no criteria/metrics standardization for measuring the performance and specifying if it is secure, scalable or efficient enough. Thus, leading to inconsistent measures and conclusions. For example, the proposed IDSs in [21,43,44] used different performance measurements, although they were used for the same purpose and for the same attack and using the same communication protocol. To clarify more, a performance comparison between those IDSs is provided in Table 1. The compared IDSs were developed with the same properties:

- Goal and Purpose: Energy Efficient Attack Detection (or Detect the attack with the least amount of consumed energy).
- Detected Attack: Packet-Dropping Attack
- Used Communication Protocol: LEACH protocol

However, other important measurements should have been employed that are more relevant to the packet-dropping attack. For example, number of dropped packets, false positive (any normal behavior that is identified as anomalous or malicious) and false negative (any malicious behavior that is identified as normal) ratios. As for the energy efficiency, it was important to know when did the first and last node die throughout the simulation time.

In order to develop and test IDSs, SE processes are required, starting by the first step which is selecting the suitable software model. In [45], a recent comparative analysis was provided to show the differences between the SDLC models. The features that were used to conduct this comparative analysis include but are not limited to: requirement specification and understanding, resource and cost control, risk involvement, analysis, and reusability. In addition, in [46], another comparative analysis was provided, and this analysis was focused on three models, which were Waterfall, Spiral, and Incremental models. This analysis was performed through discussing the strengths, weaknesses, and suitability of the models. Thus, from the provided comparative analysis, it can be seen how some models are chosen over the others due to their properties and how they match the system’s requirements. Each model consists of set of phases that provides a standard development of a system. Following such models ensures the delivery of high quality systems, manages and keeps track of risks, and prevents project failure that are caused from either not understating the requirements, poor project planning, and/or change control [47,48].

Therefore, in other words, not all requirements are identified, implemented, and satisfied. Even worse, in some cases, requirements have been also compromised. In [48,49], these problems were categorized as high software risks that may lead to software failure. However, system failure is not limited to not detecting an intrusion, as these systems provide a very critical service, which is security. Therefore, any absences, incorrect, or misuse of the system’s requirements that may cause security vulnerabilities in the system is categorized as system failure.

## 3. Research Methodology and Components

**Figure 2:**
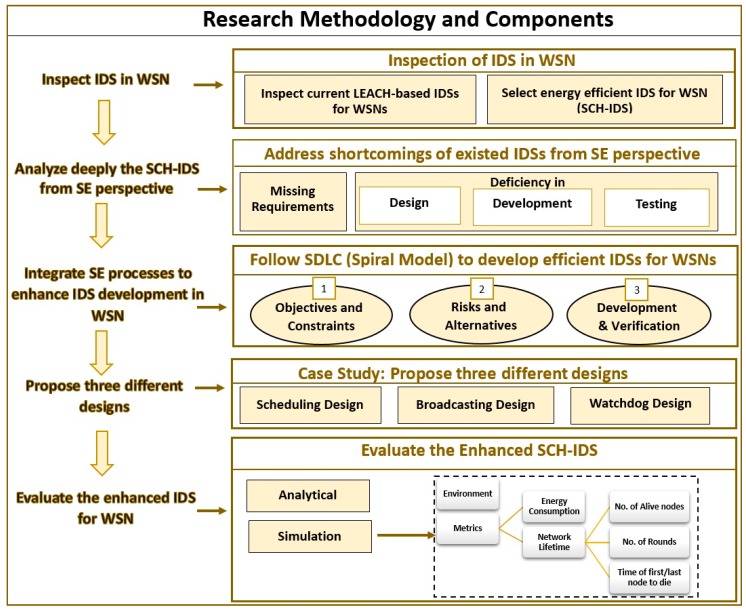
Research methodology and components.

Figure 2 illustrates the research methodology followed in this paper. The main aim of this research is to investigate the impact of applying SDLC processes on the performance of IDS in WSN. Therefore, this research started by investigating current energy efficient IDSs in WSNs. As LEACH is one of the famous and heavily used energy-efficient protocols [15,16,17,18,21,22,23,24,27,28,29,30,34,35,36,37,38,39,40,41,42,43,44] to serve the routing and communication services in WSN, consequently, IDSs built over LEACH were investigated. One of the current LEACH-based IDS (SCH-IDS) has been chosen by this study to be deeply analyzed from SE perspectives. This analysis resulted in addressing the shortcomings of the chosen SCH-IDS in regard to missing requirements and inefficient design, development, and testing processes. 

In order to show the value of applying proper SDLC to the development of IDS in the field of WSN, a study was conducted to find the best SE process model to be integrated with the development of IDSs. Spiral model was applied including its phases: Determine objectives and constraint, identify risks and evaluate alternatives, develop and verify the system, and plan the next phase. 

To examine the influence of applying SE processes, this research focused on the design and development phases. The authors of SCH-IDS did not detail the design used to build their IDS. Therefore, three different designs were proposed to provide the defined services of the SCH-IDS. These designs were called: Scheduling, Broadcasting, and Watchdog.

After that, these designs were evaluated analytically and then implemented using a network simulator. The evaluation metrics used were the energy consumption and the network life time. The consumed energy was measured in joules, whereas the network lifetime was measured using: number of alive nodes, number of rounds, and the time of the first/last node to die (in seconds).

The results of the evaluations were analyzed and then summarized. These results showed how integrating SE and IDS processes could improve the performances of IDSs, which in turn will enhance the security of the applications and systems running over different types of WSNs. 

## 4. IDS Analysis from Software Engineering Perspectives

For the purpose of illustration and discussion, this research work has taken an IDS that was developed by the authors of [21] for analysis and review from SE perspectives. The following sections will elaborate more on current IDSs in general and the chosen IDS to be specific.

### 4.1. Shortcomings of Existing IDSs from SE Perspectives

Currently developed IDSs do not show clear, structured software development processes. Consequently, resulting inadequate requirement management, processing, validation, and verification of requirements quality [19]. To elaborate more on the evidence provided in the literature, the detection process is not deployed at each layer of the hierarchy. For example, the malicious behavior can occur at the nodes layer, CHs layer, or BS layer. Thus, the problem is being unable to detect all possible malicious behaviors which affects the performance of the proposed IDSs in terms of increasing the detection delay and the energy consumption, in addition to degrading the detection accuracy, as illustrated in previous studies.

To discuss further the software failure causes that were found in the IDSs, some of them have been presented in [47,48,50] and they include but are not limited to the following:

- Poor system development planning.
- Inadequate requirement engineering process.
- Requirements not adequately identified, managed, and validated.
- Unclear and badly defined requirements.
- Incorrect requirements.
- Misunderstanding of requirements.
- Requirements continually changing.
- Not all requirements are traced while testing.

Therefore, the above points need to be considered when developing an IDS and all requirements need to be gathered, fulfilled, and traced with regard to WSN resources limitation, to be specific, energy supply limitation. In order to achieve this goal, the system development must go through a set of SE processes.

**Figure 3:**
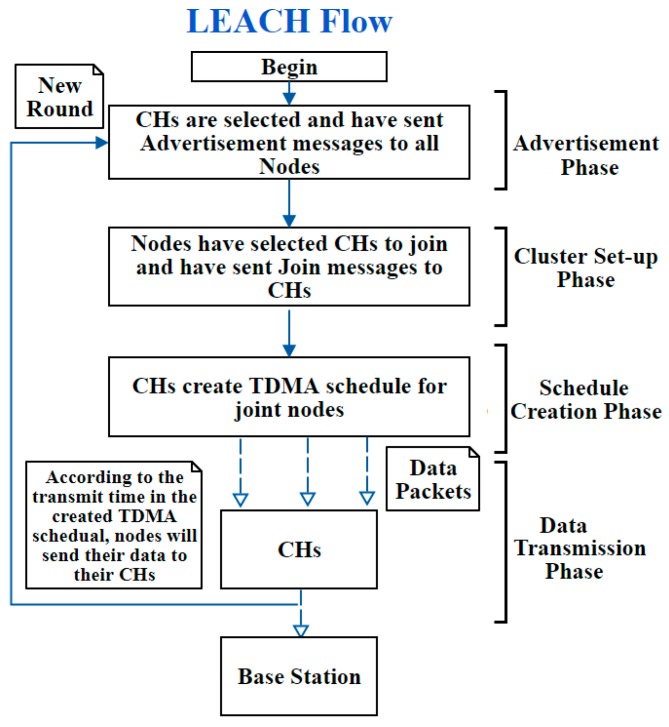
LEACH routing protocol flow.

Engineering an IDS while following the needed SDLC phases, applied on WSNs systems, is important to develop an efficient IDS—thus allowing the system to deliver the required functionalities, with respect to operational constraints, with an improved performance, accuracy, and reliability [51]. Therefore, this study has selected a hierarchical energy efficient IDS that was proposed in [21], to illustrate the lack of SE and how it is important to have a synergy between the SE field and WSN field. This IDS detects Black hole attacks, which is an attack that drops the whole data packets and prevents them from reaching the BS. In other words, dropping packets means that data will not be sent. The routing protocol used by this IDS was LEACH protocol, and this is why their IDS was energy efficient. However, the LEACH protocol has no security services; in other words, the LEACH does not detect attacks. To clarify it more, Figure 3 illustrates the LEACH routing protocol.

Therefore, on the same routing scheme of the LEACH protocol, a black hole detection schema was added. However, adding security to the LEACH is challenging because it is dynamic and it uses randomness in some of its functionalities [16]. Consequently, it periodically rearranges the CHs and changes the links between sensor nodes, due to the fact that it has a lot of overhead and hence it is not a recommended routing technique to provide security with the least amount of resource usage, as these properties make achieving security more difficult [16]. Furthermore, the detection process was performed on CHs only because LEACH protocol is a cluster-based protocol that relies essentially on CHs for data aggregation and routing [24,36,37]. Thus, electing a malicious node as CHs is the most devastating and damaging attacks to the network [36,37].

The detection schema proposed in [21] selected a second cluster head (SCH) that keeps track of what has been received by the CH. The SCH was selected based on the node that has the highest remaining energy. The tracking process starts by having the nodes, associated with the CH, sending control packets to the SCH. The control packets contain the node identifier (ID) and the number of packets sent to the CH (Nbrpk). The associated nodes send their control packets at the end of the transmission phase to the SCH. Then, the SCH sends its received data to the BS. Afterwards, the BS will compare what it has received from the CHs and SCHs and accordingly decide if an attack has been occurred or not. The attacking cases that have been considered by the authors were on the level of CHs only.

**Figure 4:**
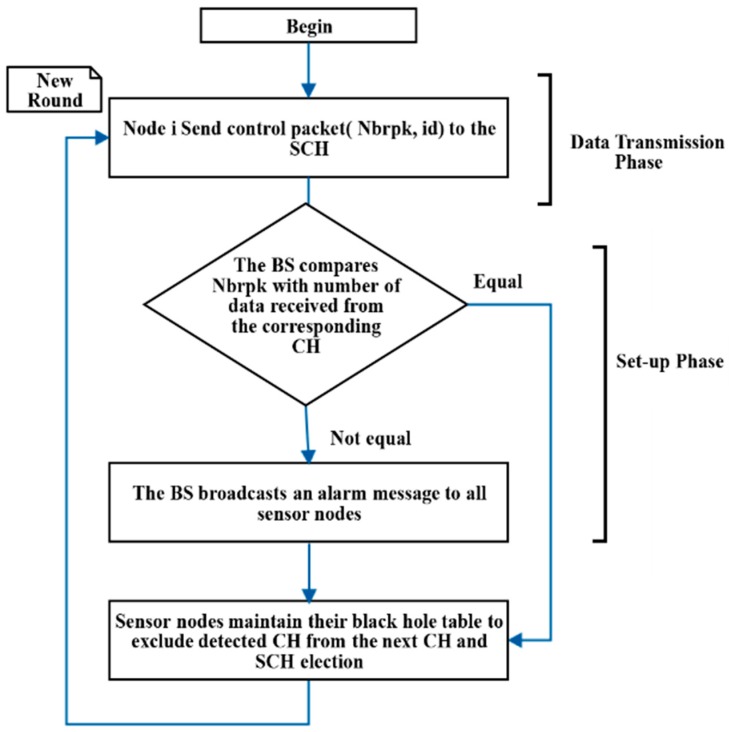
SCH-based intrusion detection scheme (SCH-IDS).

For example, if the BS gets 0 data packets from the CH and gets 10 data packets from the SCH, then this CH is determined by the BS as an attacker node. Afterwards, when the BS detects an attack, it broadcasts an alarm message to all nodes to notify them about it. Each sensor node maintains a black hole table to prevent the selection of malicious nodes as CHs in the next rounds. To clarify it more, the data flow of the authors’ proposed scheme (SCH-IDS) in [21] is illustrated in Figure 4.

However, this proposed schema have many vague steps and requirements. Thus, after an extensive analysis, we have reached a set of questions that have no answers in their paper. Listed below are the main steps and requirements that were found missing and not adequately identified:

- Who has selected the SCH? Was it the BS or the CH? And, if it was the CH, then who has informed the BS with the identity of the SCH?
- How was BS notified as to who was the SCH?
- How were the rest of the nodes notified as to who was the SCH?
- How was the current energy calculated and based on what?
- How was the attacker dealt with? Was the attacker excluded from the network?
- How were the performance measurements calculated? In the energy consumption calculation, was the attackers’ energy included in the calculations?

Moreover, some of the SE drawbacks that were found in this proposed IDS are:

- Poor System Development Planning: What is the researchers’ plan to overcome collisions and node death (before packet delivery)? In other words:
  - What if a certain node dies before or while sending its controls packets to the SCH? This case has a high probability of happening as control packets are sent at the end of the transmission phase so the node might be dead during the round.
  - What if the SCH dies before or while sending its data to the BS?
  - What if the CH dies before or while sending its data to the BS?
- Requirements Not Adequately Validated: What is the detection approach provided if the SCH is the malicious node?
- Requirements Not Well Defined: The requirement of making the associated nodes message the SCH causes too much overhead on the network and consequently causes energy loss.
- Requirements Not Adequately Managed: The selection of the SCH was based on the current remaining energy which was easy to retrieve in simulation—however, in real life, how this could be known and guaranteed.

### 4.2. IDS Enhancements from an SE Perspective

From the illustrated drawbacks and unclear requirements, we can see that the authors’ solution was not well studied and it also did not cover all the requirements. Therefore, this study finds IDS in [21] a good case study to be examined in order to show the set of cases and requirements that have not been handled and the inefficiency of the system design due to not following the SE processes—moreover, since the authors have not defined clear requirements or system design to follow and, basically, they have left the readers with possibilities. Therefore, Section 4 presents a case study that shows the different designs that are suggested in this paper, with which the IDS could have been built upon.

To illustrate the enhancements proposed by this study, the following were added to the selected IDS:

- Add a new factor to the selection criteria of the SCH, which is the Received Signal Strength Indicator (RSSI). RSSI is defined as “a measurement of the power present in a received radio signal” [52]. Each node within the WSN has RSSI value. The radio signal strength decreases with distance [53], so it is a negative correlation between the signal strength and the distance. Therefore, from the RSSI value, we can determine the distance of the node. This factor has been added to guarantee that the chosen SCH node would be the closest node to the CH because our goal is to ensure that the SCH node can hear all in/out transmissions of the CH.
- Change the monitoring and tracking process of the SCH, by deploying the Watchdog technique instead of letting the nodes contact the SCH. The Watchdog mechanism is one of the intrusion detection techniques used in WSNs [54]. Thus, it is a monitoring technique that monitors the nodes within its range (i.e., nearby nodes) [54,55]. Once the Watchdog technique is adopted, only the CH and the BS know who are the SCHs and not all nodes. This will be discussed further in the next section.

Moreover, to discuss how these enhancements improved the performance of this IDS, a theoretical analysis is provided in Table 2.

**Figure 5:**
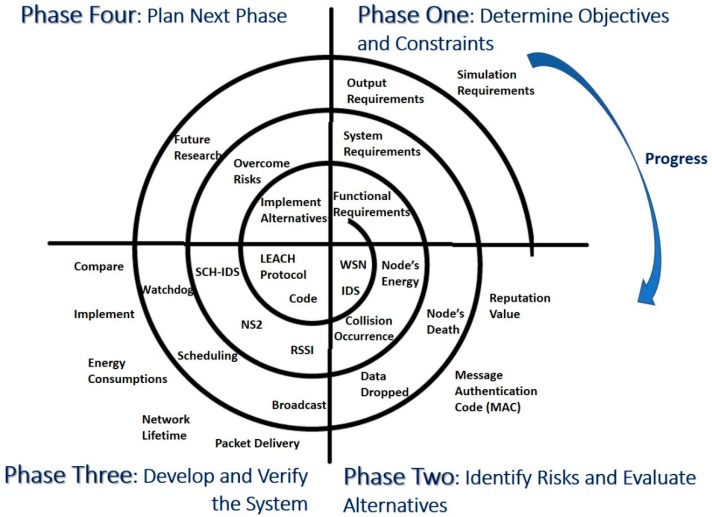
Applying the spiral model to the development of IDS in WSNs.

Although IDSs are considered a type of software security solutions, IDSs’ developers are not showing a visible software development process in their proposals. Thus, in order to implement these enhancements and develop an enhanced version of the discussed IDS, this research has started with the first and very important step, which is to select the correct and suitable SDLC model. The selection process depends on a set of factors called the selection factors, as mentioned in [56]. However, to fulfil the characteristics of developing an IDS, the factors in [56] are not enough. Therefore, according to the factors in [56] and the ones in [57,58], a combination of factors have been created and then applied to the characteristics of developing an IDS [6,9,22]. Accordingly, it can be decided that the suitable model is the Spiral Model. The spiral model of a software process is broken down into four phases. By going through the model phases (Figure 5), it is shown how the development of the proposed IDS was accomplished. The details are illustrated below:

(1) Phase One: Determine Objectives and Constraints 

The first stage is to identify and collect the system’s objectives and requirements. It is the most important and critical stage of the development process [56,57] because any requirement that is not resolved at this stage will be carried out through the rest of the SDLC.

Therefore, requirements have been gathered and analyzed from the beginning to cover all requirements in the IDS hierarchy layers, with regard to resources constraints. Firstly, in reference to the categories defined in [6,59,60], the general and main requirements are listed in Table 3.

(2) Phase Two: Identify Risks and Evaluate Alternatives 

The second stage is to identify the alternatives and risks and evaluate them. Risks are definite in IDSs; therefore, the risks associated with the enhanced IDS and their effects are illustrated in Table 4.

Thus, from the discussed risks, it can be seen that the main effect is the probability of false-positive detections. As for the alternatives, they are defined and discussed in Table 5.

(3) Phase Three: Develop and Verify the System

In order to develop, verify, and test the IDS, this paper used the network simulator tool version 2 (NS2) [61], which is well known and widely used in the field of network simulation. To get high reliability, accuracy, and efficiency in the developed IDS, this study has performed two steps before starting the actual development of the enhanced IDS:

Step One: Study the LEACH protocol implementation

The LEACH code is an open source code that was developed by a couple of researchers from the Massachusetts Institute of Technology (MIT). This was a good starting point for requirement engineering as well as exploring and learning the behavior of the WSNs.

Step Two: Implement the compared-to approach [SCH-IDS] developed in [21]

The source code of this IDS is not an open source code; therefore, we had to implement the IDS scheme but SCH-IDS had a lot of unclear points as mentioned before. Thus, in this research, three different designs were suggested, implemented, and tested. 

In spite of that, this has offered a chance to observe the different aspects that must be considered when implementing the requirements. Furthermore, we were able to find another contribution in the SE field, which is how different software designs of IDSs can affect the WSN performance. The details of how the development was carried out and tested are also described in Section 5 and Section 6.

(4) Phase Four: Plan Next Phase

In this phase, we plan to overcome the risks and implement the suggested alternatives and explore their effects. This phase could be considered as future research work and extension to this research study.

## 5. Case Study: Propose Three Different Designs

This section illustrates the three different software designs that were suggested for the selected IDS (SCH-IDS), to show how different software designs can affect the network performance.

A. First Design: Scheduling Design

**Figure 6:**
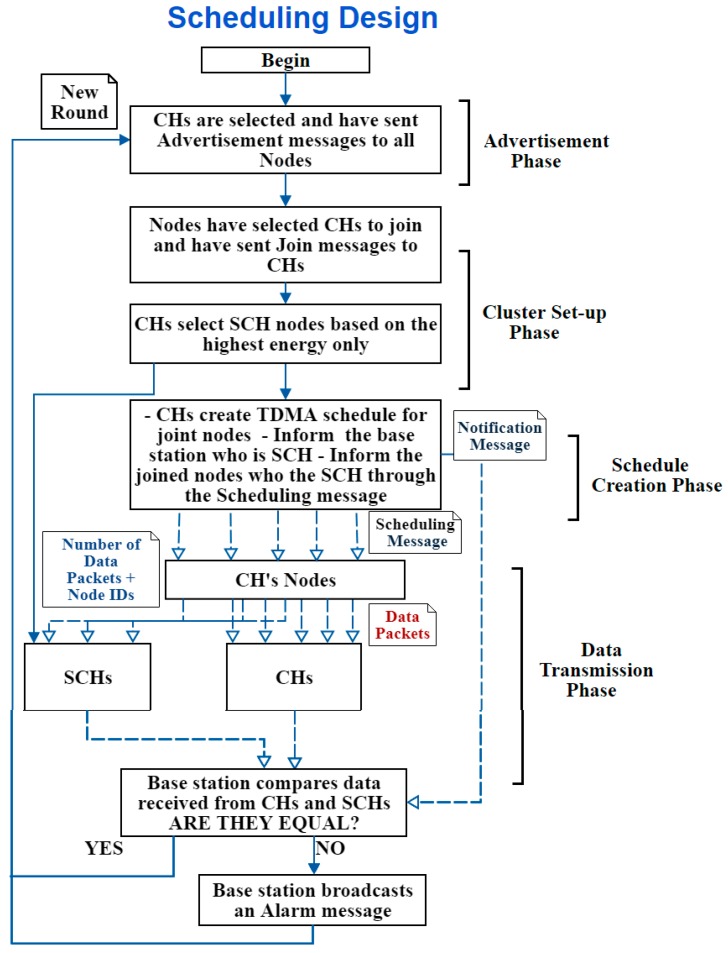
Scheduling design process.

This design works by informing the nodes whom the SCHs are—through the scheduling message sent at the “Schedule Creation Phase” in the original LEACH protocol; therefore, it is called the Scheduling design. Thus, in addition to the data usually sent in the scheduling message, extra data is added to it, which is the identity of the SCH node. In other words, no extra messages are required to send this information. The message is sent by the CHs to their joined (i.e., associated) nodes, so the nodes only know the SCH of their CH. To clarify it more, Figure 6 illustrates the scheduling design.

B. Second Design: Broadcasting Design

**Figure 7:**
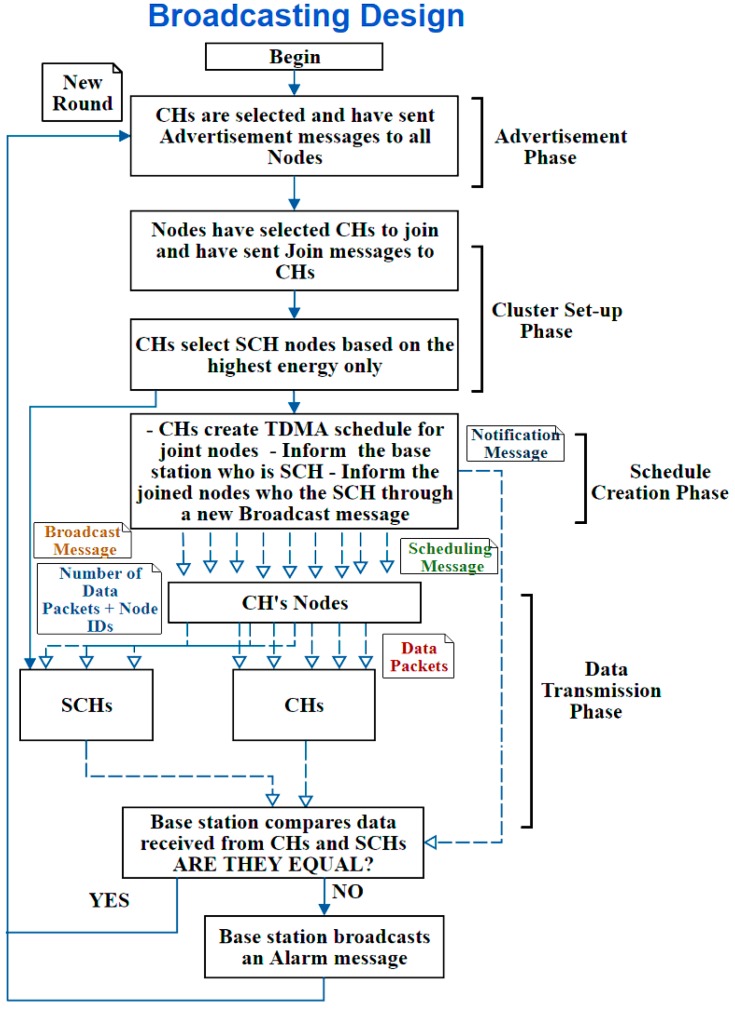
Broadcast design process.

In this design, the nodes are informed whom the SCHs are through a new broadcast message sent during the “Schedule Creation Phase”; therefore, it is called Broadcasting design. Thus, a new message type has been introduced in this design, which the “SCH Broadcast Message”. The message is sent by the CHs to their joined nodes, so the nodes only know the SCH of their CH. To clarify it more, Figure 7 illustrates the broadcast design.

C. Third Design: Watchdog Design

This design is the third design that was developed in this study. The SCH nodes are selected as watchdog nodes at the “Schedule Creation Phase” to monitor CHs. Therefore, it is called the Watchdog design. SCH can monitor the CH because it is located within the CH’s range (nearby node). The selection criteria of the SCH were mainly based on the Received Signal Strength Indicator (RSSI). This way, the closest node to the CH with the highest remaining energy will be selected as SCH. Watchdog SCH can listen to all sent and received packets from/to the CH. Therefore, this SCH is able to submit all monitored data to the BS which will compare them with the data received by CH to detect whether this CH is a benign node or an attacker. 

**Figure 8:**
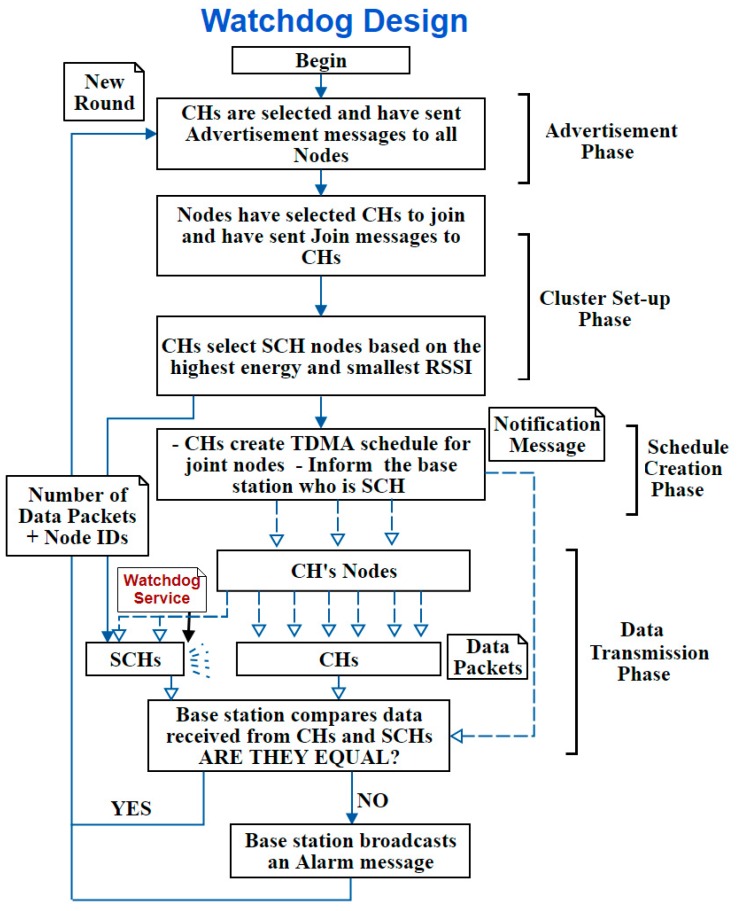
Watchdog design process.

This design deploys the monitoring technique instead of the message passing technique. Thus, the nodes are not informed whom the SCH is and only the CH knows its identity without sending extra messages. To clarify it more, Figure 8 illustrates the process flow of this design.

## 6. Results and Analysis

This section examines the impact of the three software designs on the performance of the selected IDS in terms of:

- Energy Consumption: This metric is defined as the amount of energy used and spent by the sensor nodes in WSN. The unit measurement is in Joules (j).
- Network Lifetime: This metric is defined as the amount of time a WSN would be fully operative. The unit measurement used here is in seconds (s). It is measured through a set of parameters, which include:
  - Number of Nodes Alive: This metric is defined as the amount of nodes that are still alive and have the energy to function.
  - Number of Rounds: Since the measured IDS is based on LEACH protocol, the operation of LEACH is divided into rounds (i.e., rounds are basically time stamps) [23]. Thus, this metric is defined as the amount of rounds that were performed in WSN.
  - Time of First Node to Die: This metric is defined as the time until the first sensor node runs out of energy in WSN.

Packet Delivery Ratio (PD): In general, sensor nodes consume most of their energy in sending and receiving packets (communication cost) [4,6,7,13,17,18,62,63,64]. Heinzelman et al. [23] demonstrated that a node needs *ETx*(*k,d*) to send *k* bits message to a destination at distance *d*, as shown in Equation (1):

(1) $$SendingCost\;\left(k,\;d\right)=\;ETx\;\left(k,\;d\right)\;=\;Eelec*k\;+\;Eamp*k\;*\;d^{2}$$

where *Eelec* = 50 nJ/bit, *Eamp* = 100 pJ/bit/m2.

Additionally, a node needs *ERx(k)* to receive a *k* bits message—Equation (2):

(2) receivingCost (k) = ERx (k) = Eelec∗k

in case of sending and receiving only one packet. However, to calculate the overall cost, the total number of packets (Sent packets and Received packets) need to be considered. Thus, the total cost is calculated in Equation (3):

(3) $$Overall\;cost\;=\;\sum_{i=1}^{Spkt}ETx(ki,di)+\sum_{j=1}^{Rpkt}ERx\left(kj\right)$$

where *Spkt* is total number of sent packets and *Rpkt* is total number of received packets.

Therefore, two main factors are affecting the amount of consumed energy—the number of packets and the size of the packet itself (in bits) as will be illustrated in the results below.

The different designs of the IDS were developed and tested using the NS2.34 simulation tool [64]. For the purpose of removing the effect of randomness caused by the simulation environment, each experiment was repeated many times and then the results were averaged.

Moreover, this takes us to another problem, which is the lack of systematic tools used by engineers to check if the requirements are achieved [33]. In addition, there are no available standard IDS test suites [59], so engineers need to generate both malicious activities and benign activities to test their IDSs. 

The simulation parameters that were used in the testing process are summarized in Table 6. In addition, Table 7 lists the notations used in the following equations and their meanings.

**Figure 9:**
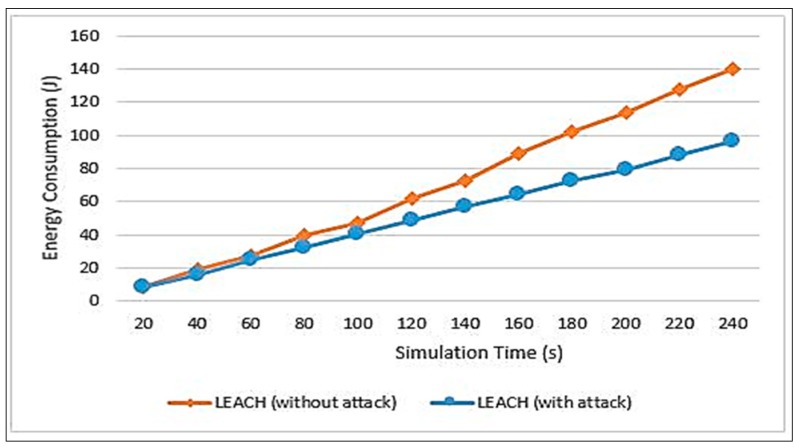
Energy consumption measurement.

**Figure 10:**
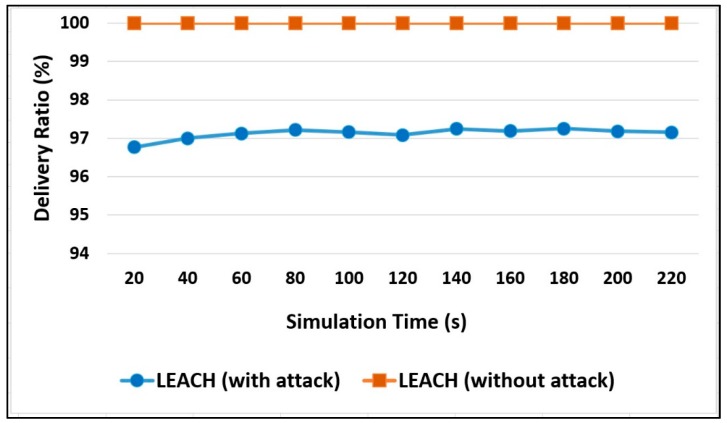
Packet delivery ratio measurement.

**Figure 11:**
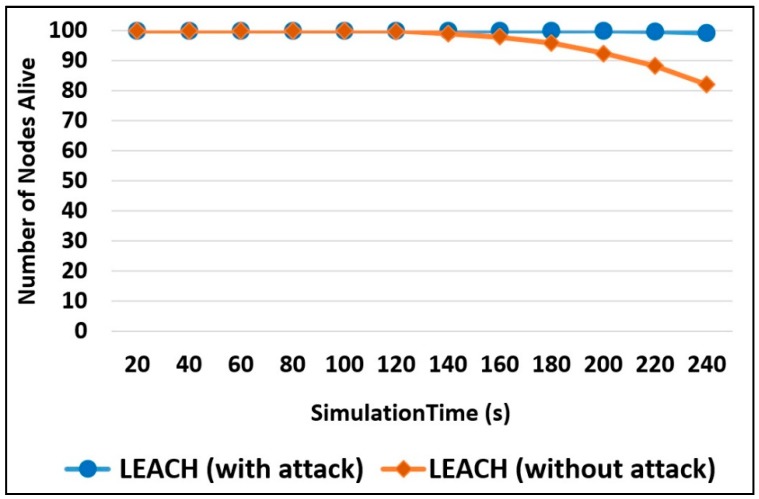
Number of nodes alive measurement.

This research has studied the effect of black hole attacks before being detected and excluded from the network. Two scenarios were performed on the original LEACH, the first one is the normal case without any attacks and the second one is after injecting the attack. The results of implementing these two scenarios are illustrated in Figure 9, Figure 10, and Figure 11. These results are after injecting 30% of the network’s nodes with packet-dropping attacks. Figure 9 illustrates the amount of consumed energy along the simulation time. It can be seen that the consumed energy within an attack is less than without an attack. This is because, when the node drops data packets, it basically does not process or transmit data and hence it saves energy. On the other hand, this has a bad impact on the packet delivery ratio as will be explained in Figure 10.

In reference to [65] and in case of the no attack scenario, it has been proven that the amount of sensed data packets that are delivered to the BS at the end of each round is calculated in Equation (4):

(4) $$Sent\;Senesed\;data\;\left[No\;attack\right]=\;\overset{NC}{\underset{i=1}{\sum }}\frac{\mathrm{NO}-\mathrm{DATA}-\mathrm{PKT}\;}{\;CM\;of\;CHi}\;$$

According to LEACH, CH receives the sensed data from the sensors nodes/cluster members (CMs) according to the TDMA schedule, it aggregates them into one packet, and sends it to the BS. Throughout the round, the number of packets sent to the CH from CMs is (NO-DATA-PKT), but, due to the aggregation process, only ($\frac{\mathrm{NO}-\mathrm{DATA}-\mathrm{PKT}}{CMs\;of\;CHi})$ packets will be sent to the BS. Having NC of CHs, then the overall data packets received by BS are $\;\sum_{i=1}^{NC}\frac{\mathrm{NO}-\mathrm{DATA}-\mathrm{PKT}}{\;CMs\;of\;CHi}$.

However, in case of compromised CHs, which will drop all packets received by them, the number of sent packets will be reduced as calculated in Equation (5), which explains the reduction in the consumed energy with the existence of packet dropping attacks. Sent Senesed data [With attack]=

(5) $$\overset{NC}{\underset{i=1}{\sum }}\frac{NO-DATA-PKT}{CMs\;of\;CHi}\;-\;\overset{NC^{\prime }}{\underset{j=1}{\sum }}\frac{NO-DATA-PKT}{CMs\;of\;CH^{\prime }j}.$$

Furthermore, the effect of the attack was measured through the packet delivery ratio because what this attack basically does is drop the packets, so it is important to measure the amount of delivered data. Packet delivery ratio is defined in Equation (6), which is the ratio of the number of delivered data packets to the destination to those generated by the source [66]:

(6) $$PDR=\;\frac{No.\;of\;PKT\;Delivered}{No.\;of\;PKT\;Sent}.$$

Therefore, in Figure 10, it can be seen that, without an attack, the data delivery ratio is 100% because all data sent are received. On the other hand, the percentage has decreased when the attacks were injected. The percentage of the delivery ratio has reached 97% at a simulation time of 220 s, which will continue to decrease throughout the network lifetime.

As shown in Figure 11, with the existence of security attacks, the nodes live longer in the network as compared to the network that has no attacks. On the other hand, the percentage has decreased when no attacks were injected to drop the sent packets. The percentage of alive nodes has reached 77% at simulation time 240 s. 

Afterwards, the results of the implementation and testing of the suggested designs are compared and discussed in the following subsections. 

### 6.1. Energy Consumption 

**Figure 12:**
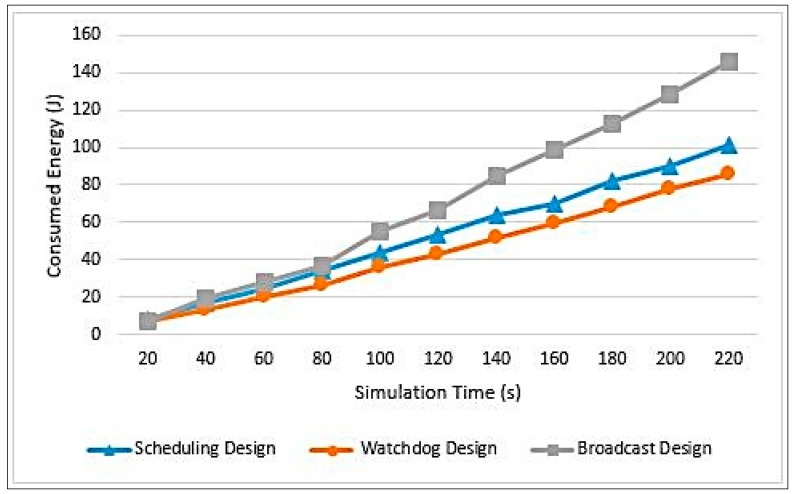
Energy consumption measurements.

The more energy the network’s nodes have, the higher the probability of detecting an attack and the longer the network will live to perform its services. Figure 12 illustrates a comparison between the three designs in terms of energy consumption.

As can be seen in Figure 12, the Watchdog design has consumed the least energy among other designs. This is due to the Watchdog monitoring mechanism, as it has reduced the number of message transmissions required and hence it has reduced the energy cost. The Watchdog technique has eliminated the need to have communication between CHs with their associated nodes and between SCHs and the associated nodes of the monitored CHs. The most expensive design is the Broadcasting design. This is due to the message transmission process performed at the “Schedule Creation Phase” and “Data Transmission Phase”. This design is exactly the opposite of the Watchdog design. As for the Scheduling design, it is more similar to the Broadcast design than the Watchdog design. The only difference is that the CH uses the same scheduling message used in the “Schedule Creation Phase”, however with small extra data added to it. Thus, this design does not initiate a new message transmission to inform the associated nodes with the identity of the SCH. It only increases the message size. Equations (7)–(9) provide analytical analysis for the cost of the three designs only in the “Schedule Creation Phase”.

Equation (7) calculates the cost in the case of Scheduling design after adding the identity of the second cluster head ((SCH-BS)_MSG_) to the schedule message (TDMA_MSG_). This addition increases the message size and consequently increases the transmission cost. Scheduling Design Cost =

(7) $${\left(SCH-BS\right)}_{MSG}+\overset{NC}{\underset{i=1}{\sum }}CNi\left[TDMA_{MSG}+SCH_{MSG}\right]$$

Equation (8) shows the new message added by the Broadcasting design (SCH_MSG_). This new message with the new added headers will be sent to all cluster nodes which will cost even more energy. Broadcasting Design Cost =

(8) $${\left(SCH-BS\right)}_{MSG}+\overset{NC}{\underset{i=1}{\sum }}CNi\left[TDMA_{MSG}\right]+\;\overset{NC}{\underset{i=1}{\sum }}CNi\left[SCH_{MSG}\right]$$

Equation (9) shows the original messages initiated by LEACH. Only the message sent to the base station is generated by this design, which is a common message in all three proposed designs: Watchtog Design Cost =

(9) $${\left(SCH-BS\right)}_{MSG}+\overset{NC}{\underset{i=1}{\sum }}CNi\left[TDMA_{MSG}\right]$$

### 6.2. Network Lifetime

**Figure 13:**
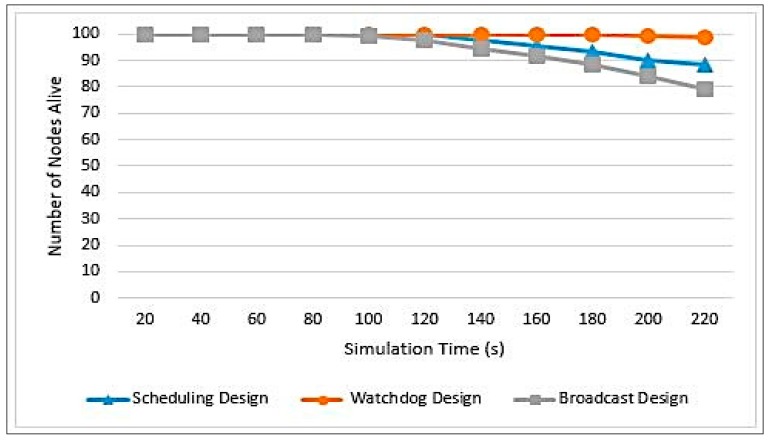
Number of nodes alive measurements.

Network lifetime is measured through a set of parameters, which include:

Number of Alive Nodes: The number of nodes alive in the network is an indicator for the network lifetime because, as long as there are functioning nodes, the network will keep running. Figure 13 illustrates a comparison among the three designs in terms of the number of alive nodes. It can be seen that, in Watchdog design, the nodes start to die after a long period of time when compared to the rest of the designs. Thus, this design will increase the network lifetime. This is due to the same reasons explained in Figure 11. The least efficient design is the Broadcasting, and this is due to the message transmission process done at the end of each round and mainly because of the SCH notification process, where each CH broadcasts a message to the entire associated nodes, to inform them with the identity of the SCH. Routing messages are very expensive in WSNs and their cost can be observed by this design.

Number of Rounds: The more rounds in the network, the longer the network will live and the more services will be provided and hence the higher probability of user satisfaction. Table 8 illustrates a comparison between the three designs.

Table 8 stresses the inefficiency of Broadcast design as it performs the least number of rounds among Scheduling and Watchdog designs which reported a close number of rounds.

Time of First Node to Die: The earlier the node dies, the more energy it has been consumed and the less time for the network to last. Table 9 illustrates a comparison between the three designs in regard to this metric.

It can be noticed that the earliest first node to die was in the Broadcast design. This indicates the shortening in the network lifetime, which means that the network did not live for long here. Consequently, Broadcast design is the worst design among them all. On the other hand, the Watchdog design had the latest first node to die, which outperformed the other two designs. Table 10 summarizes the results of the overall tests which were carried out.

**Figure 14:**
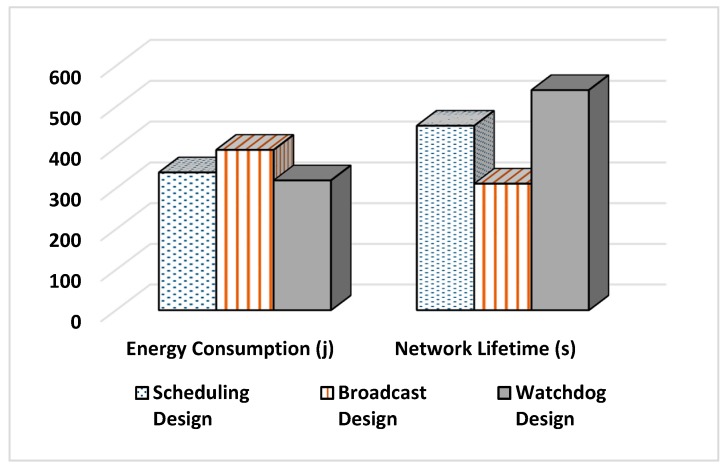
Results summary.

Figure 14 clarifies the comparison between the network lifetime and consumed energy.

To conclude, the Watchdog design was the best design among the three suggested designs. The Scheduling design comes afterwards and then the Broadcast design. This is due to the excessive number of message transmissions, especially in the Broadcast design, causing the nodes to lose their energy over message passing.

As can be also observed from the above results, this research highlighted the importance of following proper SDLC process while developing IDS to ensure efficient, secure services running over WSNs.

Since all packets can be tracked and logged within an IDS, it is very important to utilize those logs. Those logs are used for the purpose of information gathering, monitoring, and analysis. 

**Figure 15:**
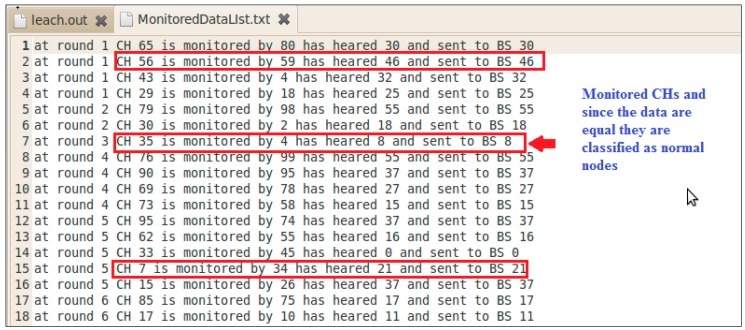
Monitoring report.

**Figure 16:**
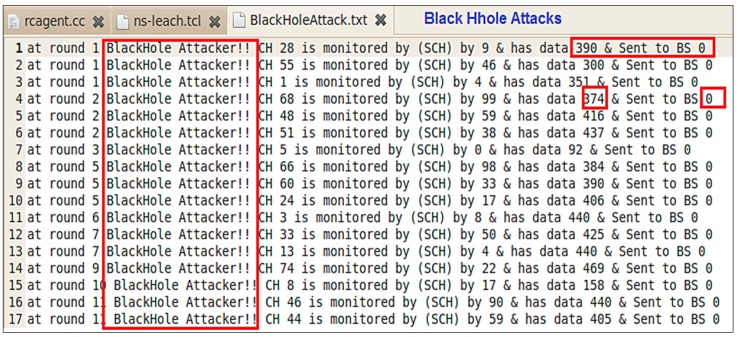
Detection report.

Figure 15 illustrates the “Monitoring Report” that collects all sorts of data attributes and Figure 16 shows the “Detection Report” that has all the detected Black hole attacks. This concludes the efficiency of detecting Blackhole attacks while preserving the network resources after following the SE processes in building the IDS for WSN.

Overall, these research results confirm that not following proper SE processes while developing intrusion detection systems for WSNs will:

- make it difficult for the researchers/developers to get sufficient details about pervious/existing systems to be fully re-implemented. This is for the purpose of achieving accurate comparisons with them, especially when the source codes of their solutions are not provided.
- miss important phases in the development process which consequently affects the behavior and the performance of their systems and their provided services.

At the same time, it is challenging to ensure that researchers/developers proposing and implementing IDS for WSN are having proper software engineering background to perform correct integration of both fields. 

## 7. Conclusions and Future Work

This paper explored the development of an energy efficient IDS for packet-dropping attacks in WSNs through following the SDLC phases, processes, and techniques. Moreover, this paper studied the absence of the SE practices and their effect on the overall results and in the development process such as missing requirements and inconsistency in the testing process and measures.

This research started by presenting the methodology followed in this research. The purpose of this research is mainly to address the lack of SE practices applied in the field of WSNs in general and in the development of IDSs in particular. Applying proper SE process models while developing IDSs for WSNs will provide efficient, accurate detection services for security attacks in their applications.

An IDS for WSN was chosen to analyze the missing SE phases in the current IDSs. Then, the SE Spiral model with its four phases: “Determine Objectives and Constraint”, “Identify Risks and Alternatives, “Develop and Verify”, and “Plan Next phase”, was proposed and applied. Consequently, this study developed a new enhanced version of an IDS through following the SDLC phases [Spiral model] to detect Blackhole attacks with high efficiency in terms of energy consumption and network lifetime. 

To explore the SE need more, three different IDS designs were proposed to illustrate the effect of software design, development, and testing on the IDS performance results. Then, the developed IDSs were tested and validated through simulation modeling using NS2 simulator. Lastly, conclusions were drawn from the results’ comparisons and analyses performed. The results of the three suggested designs: Scheduling, Broadcasting, and Watchdog, with respect to the evaluation metrics, revealed that Watchdog design was the best design among the three suggested designs. The Scheduling design comes afterwards and then the Broadcast design. The Broadcast design was an inefficient design as it has a lot of message transmission overhead, causing the nodes to lose their energy over message passing. 

In terms of energy consumption, the Watchdog succeeded to save up to 20% of the network energy more than the other two designs. Moreover, it prolonged the network lifetime by increasing the time of the first node to die by 15.5% and 66.87% in comparison to Scheduling and Broadcasting designs, respectively. Additionally, Watchdog increased the time of last node to die by up to 73% compared to Broadcasting design.

This shows how effective considering and deploying the SE processes is while developing new or enhanced IDSs—not only to enhance the performance of the IDSs, but also to help other researchers and developers get enough details to understand how current IDSs were designed, implemented, and tested to reuse them and to have a fair comparison with them. This was one of the main contributions that this research has participated in and accomplished.

In addition to all IDS enhancements mentioned in this study, there are still more valuable enhancements that are important to mention as further research including:

- Performing extensive performance analysis on different test scenarios, such as considering external intruders, larger sample size of WSNs, and advanced attackers that have more energy than normal nodes.
- Enhancing the detection process by making it more scalable to detect Black hole attacks at the SCH level.
- Detecting other types of attacks such as Grayhole attacks.
- Eliminating all network collisions to prevent triggering false positive alarms throughout the attack detection process.

## Figures and Tables

**Table 1:** Performance Measurements Comparison.

Intrusion Detection System	Used Performance Measurements
Hierarchical Energy Efficient Intrusion Detection System [21]	Number of data packets sent to BS vs. Simulation time Amount of consumed energy (i.e. power usage) vs. Simulation time
Comparing the Impact of Black hole and Grayhole Attack [43]	Network lifetime vs. Number of nodes Number of data packets sent to BS vs. Number of nodes Amount of energy consumed (i.e. power usage) vs. Number of nodes
Selective Forwarding Attack in LEACH [44]	Number of malicious nodes vs. Number of data packets sent to BS Number of malicious nodes vs. Packet delivery ratio

**Table 2:** Analysis of algorithm’s enhancements.

Before Enhancement	After Enhancement
The monitoring process was based on letting the nodes, which are joined to CHs, communicate with the SCH and send their control packets. This has increased the number of sent messages from each node, which is expensive on the node especially if the SCH is far. Moreover, this process is performed at the transmission phase of each round.	Change the monitoring and tracking process of the SCH, by deploying the Watchdog technique instead of letting the nodes communicate with the SCH. This will decrease the energy consumption and hence increase the network lifetime. This is because the number of sent messages (overhead) has been decreased for each node and consequently saves the nodes’ energy.
The SCH selection criteria were based on the remaining energy indicator only.	Add a new factor to the selection criteria of the SCH, which is the Received Signal Strength Indicator (RSSI). This way, the closest node with the highest remaining energy will be selected as SCH. This will enhance the security and energy consumption and hence enhance the network lifetime because: It ensures that the SCH hears all the nodes that are joined to the CH. Thus, it ensures detection accuracy. In addition, the process of listening to the nodes consumes a little amount of energy however selecting the closest one will decrease this value. Therefore, this factor will contribute in increasing the network’s lifetime.
All the nodes in the network know who are the SCHs of their CHs.	Since the Watchdog technique is adopted, only the CH and the BS know who are the SCHs and not all nodes. This increases the security by having fewer nodes targeting the SCHs for an attack.

**Table 3:** Determine objectives and constraints.

Type	Requirement
Functional Requirements	The system shall: Detect intrusions, of type Black hole attacks. Be energy efficient.
System Requirements	The system shall: Not introduce new weaknesses. Use little system resources (e.g. energy). Not degrade the overall system performance by introducing overheads. Be reliable and scalable. Add a third layer to the LEACH hierarchy, called the second cluster head layer. Allow the SCHs to listen and monitor the CHs, by deploying the Watchdog technique. Allow the SCHs to communicate with the BS. Detect packet-dropping attacks at the CH layers only. Select the SCH nodes based on the highest energy indicator (EI) and received signal strength indicator (RSSI) of a node. Detect internal (within the network) intruders/attackers. Allow the BS to broadcast an alarm message when attacks occur. Maintain Black hole table for each node to prevent the selection of malicious nodes as CHs or SCHs. Automatically record actions and incidents when they occur.
Output Requirements	The system shall: Generate trace file report to log all actions. Generate monitoring report to track intrusion incidents and discover Black hole attacks. Generate network performance reports
Simulation Requirements	The functional requirements must meet real-time requirements and reflect the real-time cases and characteristics of nodes in WSNs. For example, if the IDS scheme functions based on the location of the node then it needs to consider that this consumes a lot of energy, since in real-time you will have to add a GPS to the node but in simulation the location can be easily calculated. Therefore, the developer will consider this when calculating the energy of the node in the simulation.

**Table 4:** Identified risks.

Risk	Effect on IDS
The probability of consuming and compromising the node’s energy.	The node will die and become useless. In addition, it might cause false-positive detections. For example, in the case of a CH, data will not be sent because the node died and accordingly the BS will classify this CH as a malicious node although it is not.
The probability of a collision to happen when the CHs send their data to the BS.	The CH’s data will be dropped because of the collision and accordingly the BS will classify this CH as a malicious node.

**Table 5:** Identified alternatives.

Alternative	Evaluation
Add a third selection criteria which is based on the history (reputation value) of the nodes like: has it been selected as a SCH or a CH before?	This will increase the energy efficiency of CHs and SCHs. As previously selected, CHs or SCHs have already consumed more energy than other nodes who have not been selected. This is because CHs and SCHs perform more expensive operations, including communications with the BS and processing all packet received by CH. Therefore, the non-previously selected nodes will have more energy and thus have less probability to die before previously selected nodes.
Add Message Authentication Code (MAC) for integrity and authentication process.	This will consume more energy and nodes might die sooner. However, it will increase the security and increase the originality of the node and assure authenticity.

**Table 6:** Simulation parameters.

Parameter	Value
Network surface	1000 m^2^
BS location	(50,181)
Number of nodes	100 nodes
Number of clusters	5
Size of data packet	500 bytes
Size of packet header	25 bytes
Routing protocol	LEACH
MAC protocol	CSMA/TDMA [Carrier Sense Multiple Access/Time Division Multiple Access]
Simulation time (in seconds)	3600
Initial energy (in joule)	2
Attackers’ intensities	30%

**Table 7:** Equations’ notations and their meanings.

Notation	Meaning
N	Network size
BS	Base Station
CH	Cluster Head
CH’	Compromised Cluster Heads
CN	Cluster Node
CM	Cluster Members
NC	Number of cluster heads within a round
NC’	Number of compromised CHs [attackers] within a specific round
NO-DATA-PKT	Number of data packets received by a CH
SCH	Second Cluster Head

**Table 8:** Number of rounds.

Design	Number of Rounds
Scheduling	29
Broadcast	20
Watchdog	27

**Table 9:** First node to die.

Design	Time of First Node to Die
Scheduling	330.6
Broadcast	228.825
Watchdog	381.85

**Table 10:** Results summary.

Performance Measurements	Scheduling Design	Broadcast Design	Watchdog Design
Energy Consumption (j)	339.05	394.3	319.6
Network Lifetime (s) [last node to die]	453.5	311.4	540.6
Nodes Alive at End of Simulation (No.)	4	4	4
Time of First Node to Die (s)	330.6	228.825	381.85

## Abbreviation

This appendix lists all abbreviations listed in the paper and their meanings.

Abbreviation	Meaning
BS	Base Station
CH	Cluster Head
CH’	Compromised Cluster Heads
CM	Cluster Members
CN	Cluster Node
CSMA/TDMA	Carrier Sense Multiple Access/Time Division Multiple Access
d	Distance
EI	Energy Indicator
IDSs	Intrusion Detection Systems
IR	Intrusion Ratio
J	Joules
k	Message Bits
LEACH	Low-Energy Adaptive Clustering Hierarchy
MAC	Message Authentication Code
MIT	Massachusetts Institute of Technology
N	Network Size
Nbrpk	Number of Packets Sent to The CH
NC	Number of Cluster Heads Within A Round
NC’	Number of Compromised CHs [Attackers] Within A Specific Round
NO-DATA-PKT	Number of Data Packets Received by a CH
NS2	Network Simulator Tool Version 2
PDR	Packet Delivery Ratio
PKT	Packet
RPK	Random Pairwise Keys
RSSI	Received Signal Strength Indicator
S	Seconds
SCH	Second Cluster Head
SE	Software Engineering
SDLC	Software Development Life Cycle
TDMA	Time Division Multiple Access
WSNs	Wireless Sensor Networks

## References

[1] Mohanani R., Salman I., Turhan B., Rodríguez P., Ralph P. (2018). Cognitive biases in software engineering: A systematic mapping study. IEEE Trans. Softw. Eng..

[2] Pressman R.S. (2009). Software Engineering: A Practioner’s Approach.

[3] Afanasov M., Mottola L., Ghezzi C. (2018). Software adaptation in wireless sensor networks. ACM Trans. Auton. Adapt. Syst..

[4] Akyildiz I., Su W., Sankarasubramaniam Y., Cayirci E. (2002). Wireless Sensor Networks: A Survey. Comput. Netw..

[5] Yong C., Song X., Zhao L., Yuan H., Wu G., Wang C. (2019). WSN-Based Measurement of Ion-Current Density Under High-Voltage Direct Current Transmission Lines. IEEE Access.

[6] Karray F., Jmal M., Garcia-Ortiz A., Abid M., Obeid A. (2018). A comprehensive survey on wireless sensor node hardware platforms. Comput. Netw..

[7] Almomani I., Saadeh M. (2018). S-FEAR: Secure-Fuzzy Energy Aware Routing Protocol for Wireless Sensor Networks. KSII Trans. Internet Inf. Syst..

[8] Jokhio S.H., Jokhio I.A., Kemp A.A.H. (2013). Light-Weight Framework For Security-Sensitive Wireless Sensor Networks Applications. Iet Wirel. Sens. Syst..

[9] Aley S., Kolte N. (2014). A Review On Intrusion Detection Schemes In Wireless Sensor Network. Int. J. Comput. Sci. Mob. Comput..

[10] Liu X., Abdelhakim M., Krishnamurthy P., Tipper D. (2018). Identifying malicious nodes in multihop iot networks using dual link technologies and unsupervised learning. Open J. Internet Things.

[11] Hussain R.H. (2017). A Survey on Security Challenges in Wireless Sensor Networks. J. Univ. Thi-Qar.

[12] Elhadj B., Welsh T., Hamouda W. (2018). A Critical Review of Practices and Challenges in Intrusion Detection Systems for IoT: Toward Universal and Resilient Systems. IEEE Commun. Surv. Tutor..

[13] Hamamreh R.A., Haji M.M., Qutob A.A. (2018). An Energy-Efficient Clustering Routing Protocol for WSN based on MRHC. Int. J. Digit. Inf. Wirel. Commun..

[14] Krontiris I., Dimitriou T., Giannetsos T., Mpasoukos M. (2007). Intrusion Detection of Sinkhole Attacks in Wireless Sensor Networks. Algosensors’07 Proceedings of the 3rd International Conference on Algorithmic Aspects of Wireless Sensor Networks.

[15] Ferreira A.C., Aur M. (2005). On The Security Of Cluster-Based Communication Protocols For Wireless Sensor Networks. Proceedings of the 4th International Conference On Networking.

[16] Oliveira L.B., Ferreira A., Vilac M.A., Bern M., Dahab R., Loureiro A.A.F. (2007). SecLEACH—On the Security Of Clustered Sensor Networks. J. Signal. Process..

[17] Zhang K., Wang C., Wang C. A Secure Routing Protocol For Cluster-Based Wireless Sensor Networks Using Group Key Management. Proceedings of the 4th International Conference on Wireless Communications, Networking and Mobile Computing.

[18] Qiang T., Bingwen W., Zhicheng D. Ms-Leach: A Routing Protocol Combining Multi-Hop Transmissions And Single-Hop Transmissions. Proceedings of the 2009 Pacific-Asia Conference on Circuits, Communications and Systems.

[19] Uzunov V., Fernandez B., Falkner K. (2018). Assessing and improving the quality of security methodologies for distributed systems. J. Softw. Evol. Process..

[20] Dewal P., Narula S., Jain V., Baliyan A. (2018). Security Attacks in Wireless Sensor Networks: A Survey. Cyber Security: Proceedings of CSI 2015.

[21] Athmani S., Boubiche D.E., Bilami A. Hierarchical Energy Efficient Intrusion Detection System For Black hole attacks in Wsns. Proceedings of the 2013 World Congress on Computer and Information Technology(WCCIT).

[22] Kenkre P.S., Pai A., Colaco L. (2015). Real time intrusion detection and prevention system. Proceedings of the 3rd International Conference on Frontiers of Intelligent Computing: Theory and Applications (FICTA) 2014.

[23] Heinzelman W.R., Chandrakasan A., Balakrishnan H. Energy-Efficient Communication Protocol For Wireless Microsensor Networks. Proceedings of the 33rd Hawaii International Conference on System Sciences.

[24] Wang J., Zheng L., Zhao L., Tian D. (2012). LEACH-Based Security Routing Protocol for WSNs. Advances in Computer Science and Information Engineering. Advances in Intelligent and Soft Computing.

[25] Khan K., Shiraz M., Ghafoor K.Z., Khan S., Sadiq A., Ahmed G. (2018). EE-MRP: Energy-efficient multistage routing protocol for wireless sensor networks. Wirel. Commun. Mob. Comput..

[26] Marmol F.G., Perez G.M. TRMSim-WSN, Trust and Reputation Models Simulator for Wireless Sensor Networks. Proceedings of the IEEE International Conference on Communications.

[27] Karkazis P., Papaefstathiou I., Sarakis L., Zahariadis T., Velivassaki T., Bargiotas D. Evaluation of RPL with a transmission count-efficient and trust-aware routing metric. Proceedings of the 2014 IEEE International Conference on Communications (ICC).

[28] Xiang G., Jianlin Q., Jin W. (2012). Research on Trust Model of Sensor Nodes in WSNs. Procedia Eng..

[29] Khan Y., Shah M., Khan H., Hayat N., Khan F. (2016). Amplified Forms of LEACH based Clustering Protocols for WSNs-A Survey. Int. J. Adv. Res. Comput. Eng. Technol..

[30] Pandey S., Rakesh K. (2019). Re-LEACH: An Energy-Efficient Secure Routing Protocol for Wireless Sensor Networks. International Conference on Computer Networks and Communication Technologies.

[31] Chaudhary A. (2019). Mamdani and Sugeno Fuzzy Inference Systems’ Comparison for Detection of Packet Dropping Attack in Mobile Ad Hoc Networks. Emerging Technologies in Data Mining and Information Security.

[32] Georgios S., Ibrahim M., Azer B. Sep: A Stable Election Protocol for Clustered Heterogeneous Wireless Sensor Networks. Proceedings of the Second International Workshop on Sensor and Actor Network Protocols and Applications (SANPA 2004).

[33] Mustafa S., Ali M., Hashim J. (2018). Work in Progress: Proactive Immunization Against Multiple Sink Holes in Wireless Sensor Network to Extend Its Life Span. Adv. Sci. Lett..

[34] Aslam M., Javaid N., Rahim A., Nazir U., Bibi A., Khan Z.A. Survey Of Extended Leach-Based Clustering Routing Protocols For Wireless Sensor Networks. Proceedings of the 2012 IEEE 14th Int. Conf. High Perform. Comput. Commun. 2012 IEEE 9th Int. Conf. Embed. Softw. Syst..

[35] Rahayu T.M., Lee S., Lee H. Survey On Leach-Based Security Protocols. Proceedings of the International Conference in Advanced Communication Technology (Icact).

[36] Neamatollahi P., Naghibzadeh M. (2018). Distributed unequal clustering algorithm in large-scale wireless sensor networks using fuzzy logic. J. Supercomput..

[37] Oliveira L.B., Wong H.C., Bern M., Alto P., Dahab R. Secleach—A Random Key Distribution Solution For Securing Clustered Sensor Networks. Proceedings of the 5th IEEE International Symposium on Network Computing And Applications.

[38] Abdullah L., Almomani I., Aburumman A. Secure cluster-based SIP service over Ad hoc networks. Proceedings of the 2013 IEEE Jordan Conference on Applied Electrical Engineering and Computing Technologies (AEECT).

[39] Jan B., Farman H., Javed H., Montrucchio B., Khan M., Ali S. (2017). Energy efficient hierarchical clustering approaches in wireless sensor networks: A survey. Wirel. Commun. Mob. Comput..

[40] So-In C., Udompongsuk K. (2013). Performance Evaluation Of Leach On Cluster Head Selection Techniques In Wireless Sensor Networks. Proceedings of the 9th International Conference on Computing and. Information technology.

[41] Xue Y., Lee H.S., Yang M., Kumarawadu P., Ghenniwa H.H., Shen W. (2007). Performance Evaluation Of Ns-2 Simulator For Wireless Sensor Networks. Proceedings of the 2007 Canadian Conference on Electrical and Computer Engineering.

[42] Manimozhi B., Santhi B. (2013). Comparison of Different Performance Measures of Routing Protocols in WSN. Int. J. Eng. Technol..

[43] Tripathi M., Gaur M.S., Laxmi V. (2013). Comparing The Impact Of Black Hole And Gray Hole Attack On Leach In Wsn. Procedia Manuf..

[44] Patel N.K., Singal G. (2013). Selective Forwarding Attack In Leach In Wsn. Int. J. Electron. Electr. Comput. Syst. Ijeecs.

[45] Kumar M., Rashid E. (2018). An Efficient Software Development Life cycle Model for Developing Software Project. Int. J. Educ. Manag. Eng..

[46] Alshamrani A., Bahattab A. (2015). A Comparison Between Three SDLC Models Waterfall Model, Spiral Model, Incremental/Iterative Model. Int. J. Comput. Sci..

[47] Talha M. (2018). Critical Requirements Engineering Errors Leads to Fails Software Project. Educ. Rev. USA.

[48] Pasha M., Qaiser G., Pasha U. (2018). A critical analysis of software risk management techniques in large scale systems. IEEE Access.

[49] Nicolás J., de Gea C., Nicolás B., Fernández-Alemán L., Toval A. (2018). On the risks and safeguards for requirements engineering in global software development: Systematic literature review and quantitative assessment. IEEE Access.

[50] Zahid A., Haider W., Farooq S., Abid A., Ali A. (2018). A Critical Analysis of Software Failure Causes From Project Management Perspectives. VFAST Trans. Softw. Eng..

[51] Lei J., Tian X., Zhang Z. (2018). Life Cycle and Intrusion Tolerance Optimization Topology Models for Wireless Sensor Networks. Int. J. Online Eng..

[52] Abeywickrama S., Samarasinghe T., Ho K., Yuen C. (2018). Wireless energy beamforming using received signal strength indicator feedback. IEEE Trans. Signal. Process..

[53] Wang X., Yunjian P., Lu H. An Improved Unequal Cluster-Based Routing Protocol for Energy Efficient Wireless Sensor Networks. Proceedings of the 2019 International Conference on Intelligent Transportation, Big Data & Smart City (ICITBS).

[54] Baburajan J., Prajapati J. (2014). A Review Paper On Watchdog Mechanism In Wireless Sensor Network To Eliminate False Malicious Node Detection. Int. J. Res. Eng. Technol..

[55] Terence S., Purushothaman G. (2019). A Novel Technique to Detect Malicious Packet Dropping Attacks in Wireless Sensor Networks. J. Inf. Process. Syst..

[56] Mahanti R., Neogi M.S., Bhattacharjee V. (2012). Factors Affecting the Choice of Software Life Cycle Models in the Software Industry-An Empirical Study. J. Computer Sci..

[57] Apoorva M. (2013). A Comparative Study of Different Software Development Life Cycle Models in Different Scenarios. Int. J. Adv. Res. Comput. Sci. Manag. Stud..

[58] Maheshwari S., Jain D. (2012). A Comparative Analysis of Different types of Models in Software Development Life Cycle. Int. J. Adv. Res. Comput. Sci. Softw. Eng..

[59] Scarfone K., Mell P. (2007). Guide To Intrusion Detection And Prevention Systems (Idps), Recommendations 0f the National Institute Of Standards And Technology.

[60] Metcalf T.R., Lapadula L.J. (2000). Intrusion Detection System Requirements. A Capabilities Description in Terms of the Network Monitoring and Assessment Module of CSAP21.

[61] Zhang J., Li W., Cui D., Zhao X., Yin Z. The Ns2-Based Simulation And Research On Wireless Sensor Network Route Protocol. Proceedings of the 2009 5th International Conference On Wireless Communications, Networking And Mobile Computing.

[62] Aljawawdeh H., Almomani I. Dynamic Load Balancing Protocol (DLBP) for Wireless Sensor Networks. Proceedings of the 2013 IEEE Jordan Conference on Applied Electrical Engineering and Computer Technologies.

[63] Almomani I., Saadeh M., AL-Akhras M., AlJawawdeh H. (2011). A Tree-Based Power Saving Routing Protocol for Wireless Sensor Networks. Int. J. Comput. Commun..

[64] Almomani I., Al-Kasasbeh B. Performance Analysis of LEACH protocol under Denial of Service Attacks. Proceedings of the IEEE International Conference on Information and Communication Systems (ICICS 2015).

[65] Almomani I., Al-Kasasbeh B., AL-Akhras M. (2016). WSN-DS: A Dataset for Intrusion Detection Systems in Wireless Sensor Networks. J. Sens..

[66] Gupta P., Pratyaksh G., Pranjali V., Nitin T. (2019). Reliability Factor Based AODV Protocol: Prevention of Black Hole Attack in MANET. Smart Innovations in Communication and Computational Sciences.

